# First Line CombinAtion Therapy in the Treatment of Stage II and III Hypertension (FLASH)

**DOI:** 10.3389/fcvm.2020.00046

**Published:** 2020-03-30

**Authors:** David Spirk, Sarah Noll, Michel Burnier, Stefano Rimoldi, Georg Noll, Isabella Sudano

**Affiliations:** ^1^Institute of Pharmacology, University of Bern, Bern, Switzerland; ^2^University of Zurich, Zurich, Switzerland; ^3^Service of Nephrology and Hypertension, Lausanne University Hospital, Lausanne, Switzerland; ^4^Department of Cardiology, Bern University Hospital, University of Bern, Bern, Switzerland; ^5^Heart Clinic, Clinic Hirslanden, Zurich, Switzerland; ^6^Department of Cardiology, University Heart Center, University Hospital Zurich, Zurich, Switzerland

**Keywords:** hypertension, single-pill combination therapy, hydrochlorothiazide, blood pressure, goal attainment

## Abstract

**Background:** Lowering blood pressure (BP) leads to reduced risk of stroke, myocardial infarction, and cardiovascular mortality. Single-pill combination therapies may deliver better BP control than increasing the dose of monotherapies or using more drugs separately.

**Objectives:** The aim of the present observational study was to investigate the real-life use and the effect of first line or replacement single-pill combination therapies containing irbesartan and hydrochlorothiazide (HCTZ) on systolic BP (SBP) control rate in patients with hypertension.

**Methods:** Overall, 780 patients with moderate or severe hypertension either untreated (289; 37%) or uncontrolled (491; 63%) with previous therapy were included in the “First Line CombinAtion Therapy in the Treatment of Stage II and III Hypertension” (FLASH) prospective Swiss national-wide cohort study. All recruited patients received single-pill antihypertensive combination therapy containing HCTZ and irbesartan. BP was measured at baseline and after 8-weeks follow-up according to guidelines.

**Results:** Mean reductions in office systolic/diastolic BP (SBP/DBP) were 23.7 ± 13.7/11.7 ± 8.5 mmHg, with reductions of 26.9 ± 14.1/13.0 ± 8.8 mmHg or 21.8 ± 13.1/11.0 ± 8.3 mmHg when the single-pill combination of irbesartan/HCTZ was given as first line or replacement treatment, respectively (*p* < 0.001 for differences between first line and replacement treatment in both SBP and DBP). The guidelines-recommended goals were reached in 368 (47%), 492 (63%), and 312 (40%) patients for SBP, DBP, and SBP/DBP, respectively. The SBP control rate was higher when the combination was used as first line treatment (52 vs. 44%; *p* = 0.043). Overall, 145 adverse events were recorded; hypotension in 12 (1.5%) cases, hypokalaemia in 9 (1.2%), and hyperkalaemia in 3 (0.4%).

**Conclusions:** The single-pill combination of irbesartan/HCTZ was well-tolerated and achieved substantial reductions in both systolic and diastolic BP. The SBP control rate was greater when the combination was prescribed as first line treatment as suggested by recent ESC/ESH guidelines.

## Introduction

Cardiovascular disease is the most common cause of death in the population, and hypertension is one of the most important treatable risk factors (1). Lowering blood pressure (BP) is associated with considerable benefits including a reduced risk of stroke, myocardial infarction, and cardiovascular mortality (2).

However, treating hypertension to targets is often challenging. Data from clinical studies and registries suggest that more than two-thirds of patients with hypertension require treatment with two or more antihypertensive drugs to achieve their target BP goals (3). It was shown that using a combination therapy lead to a better BP control than increasing the dose of monotherapy (4–7). Moreover, the concomitant use of drugs with different mechanisms of action can offset potential side effects of each drug (8). Taking these data into consideration, various single-pill combinations have been developed and used to improve BP goals attainment and patient adherence.

The combination of an angiotensin receptor blocker (ARB) and hydrochlorothiazide (HCTZ) is well-tolerated and able to effectively reduce BP, particularly when used as a single-pill combination. Initial treatment with the ARB irbesartan in combination with HCTZ has been proven to be as safe as monotherapy and more effective in patients with moderate and severe hypertension, in obese patients with mild-to-moderate hypertension, and in patients with type 2 diabetes and hypertension (9–14).

Swiss hypertension guidelines valid at the time when the present study was conducted recommended the use of combination therapies in all patients presenting with systolic/diastolic BP above 160/100 mmHg, even if no treatment was administered previously (15, 16). Nonetheless, hypertension remains undertreated and there is a need to implement consensus guideline recommendations in clinical practice (17). Many physicians are reluctant to treat hypertension aggressively and still prescribe low dose monotherapies for initial treatment. One reason for this cautious approach is the fear of possible adverse events, including hypotension.

In the present study, we aimed to investigate the patterns of real-life use and the effect of first line or replacement combination therapy containing HCTZ and irbesartan in terms of efficacy and safety in patients with moderate or severe (stage II or III) hypertension.

## Materials and Methods

### Study Population

In the “First Line CombinAtion Therapy in the Treatment of Stage II and III Hypertension” (FLASH) prospective national cohort study, private-practice based cardiologists and general practitioners across all regions of Switzerland were invited to enroll up to six patients per physician during a 1-year period between September 2008 and 2009. For the purpose of representativeness, Switzerland was divided into 10 geographic sectors of similar territorial and population size, and for each of these sectors, 14 physicians were randomly selected from a centralized national register and asked to participate in this study. Inclusion criteria were age ≥18 years, moderate or severe (stage II or III) hypertension either untreated or uncontrolled with previous antihypertensive therapy. All patients received single-pill antihypertensive combination therapy containing HCTZ and irbesartan; those previously untreated as first line and those previously uncontrolled as replacement treatment. There were no exclusion criteria. Written informed consent was obtained.

The study period included 8 weeks follow-up after the baseline visit. The primary endpoint was the guidelines-recommended systolic BP goal attainment of below 140 mmHg after 8 weeks of antihypertensive combination therapy.

The study was approved by the ethics committee in accordance with local regulations valid at the time of study conduct.

### Data and Definitions

Data were collected by treating physicians and entered in a standardized case report form. At the baseline visit, following parameters were recorded: patient characteristics including age, gender, weight, height, cardiovascular risk factors and co-morbidities, previous antihypertensive treatment and co-medication (if any), office systolic and diastolic BP [seated, mean of two consecutive measurements by automatic devices spaced by 1–2 min, according to guidelines (15, 18)] prior to the initiation of combination therapy containing HCTZ, and new antihypertensive combination treatment. Individual BP goals were determined according to former Swiss guidelines (<130/80 mmHg for diabetic patients and those with renal disease, and <140/90 mmHg for non-diabetic patients and those without renal disease). At the follow-up visit, office systolic and diastolic BP (seated) control, treatment tolerability including occurrence of hypotension, and compliance with study medication were recorded, and in patients not at goal a change in antihypertensive therapy was suggested.

In patients without diabetes mellitus, BP was categorized as normal (systolic 120–129 mmHg and or diastolic 80–84 mmHg), high normal (systolic 130–139 mmHg and or diastolic 85–89 mmHg), mild (stage I) hypertension (systolic 140–159 mmHg and or diastolic 90–99 mmHg), moderate (stage II) hypertension (systolic 160–179 mmHg and or diastolic 100–109 mmHg), and severe (stage III) hypertension (systolic ≥180 mmHg and or diastolic ≥110 mmHg) (15, 18). The real threshold for hypertension should have been considered as flexible and based on the total cardiovascular risk profile of each individual (18).

Clinically confirmed cardiovascular diseases were defined as myocardial infarction or coronary heart disease, heart failure, atrial fibrillation, stroke or transient ischemic attack, peripheral artery disease, and chronic kidney disease. Additional cardiovascular risk factors were defined as type I and type II diabetes mellitus, dyslipidaemia, smoking, overweight or obesity, and physical inactivity. Hypokalaemia was defined as serum potassium <3.5 mmol/l and hyperkalaemia as serum potassium >5.0 mmol/l.

First line therapy was defined as the administration of BP-lowering medication in patients with untreated hypertension at the baseline visit and replacement therapy as the use of BP-lowering medication in patients with uncontrolled hypertension at baseline despite previous antihypertensive treatment.

### Statistical Analysis

Based on the results of previous studies, we assumed that 60% of patients would attain the guidelines-recommended BP targets after 8 weeks of treatment with antihypertensive combination therapy. To reach a precision of ±5.0% of the 95% confidence interval (CI) for the estimated proportion of patients at goal, a minimum sample of 369 patients per group (first line and replacement combination therapy) was calculated.

Continuous variables with a normal distribution are presented as means with standard deviations (SD), and group comparisons were performed with the *t*-test; continuous variables with skewed distribution are described as median values with interquartile ranges (IQR), and group comparisons were performed using a rank-sum test. Discrete variables are depicted as frequencies and percentages, and group comparisons were performed with the chi square or Fisher's exact test. Data were analyzed using the STATA 10 software (STATACorp LP, College Station, Texas, USA).

## Results

### Patient Characteristics

Overall, 780 patients were enrolled by 135 participating physicians. Their mean age was 61 ± 12 (range 27–92) years, 342 (44%) patients were female, mean weight was 81 ± 16 kg, and mean height 169 ± 9 cm.

At baseline, 491 (63%) patients had uncontrolled hypertension despite prior BP-lowering therapy; of those, 158 (32%) were previously treated with an ARB, 135 (27%) with angiotensin converting enzyme-inhibitors (ACE-I), 141 (29%) with beta-blockers (BB), 89 (18%) with calcium channel-blockers (CCB), and 72 (15%) with diuretics other than HCTZ; thus, 104 (21%) patients received prior combination therapy.

Patients with uncontrolled hypertension despite previous therapy were older than those with untreated hypertension at baseline (64 ± 11 vs. 56 ± 11 years; *p* < 0.001), had more often clinically confirmed cardiovascular disease, and had more additional cardiovascular risk factors (Table 1).

**Table 1:** Clinically confirmed cardiovascular disease and additional cardiovascular risk factors in patients with uncontrolled hypertension despite previous therapy and in those with untreated hypertension at baseline.

	**Uncontrolled** ***N*** **=** **491**		**Untreated** ***N*** **=** **289**		**Total** ***N*** **=** **780**		***P***
**Established cardiovascular disease**, ***n*** (%)^*^	**206**	**(42.0)**	**38**	**13.1**	**244**	**31.3**	< 0.001
Coronary heart disease, *n* (%)	72	(14.7)	7	2.4	79	10.1	< 0.001
Peripheral artery disease, *n* (%)	36	(7.3)	5	1.7	41	5.3	0.001
Heart failure, *n* (%)	37	(7.5)	0	0.0	37	4.7	< 0.001
Atrial fibrillation, *n* (%)	36	(7.3)	1	0.3	37	4.7	< 0.001
Chronic kidney disease, *n* (%)	28	(5.7)	3	1.0	31	4.0	0.001
Stroke or transient ischemic attack, *n* (%)	27	(5.5)	2	0.7	29	3.7	0.001
**Additional cardiovascular risk factors**, ***n*** (%)^**^	**437**	**(89.0)**	**239**	**82.7**	**676**	**86.7**	0.012
Physical inactivity, *n* (%)	240	(48.9)	140	48.4	380	48.7	0.91
Dyslipidemia, *n* (%)	261	(53.2)	92	31.8	353	45.3	< 0.001
Overweight or obesity, *n* (%)	162	(33.0)	88	30.4	250	32.1	0.46
Smoking, *n* (%)	125	(25.5)	103	35.6	228	29.2	0.003
Diabetes mellitus type II, *n* (%)	146	(29.7)	40	13.8	186	23.8	< 0.001
Diabetes mellitus type I, *n* (%)	13	(2.6)	3	1.0	16	2.1	0.13

*Bold identified total cardiovascular diseases and total cardiovascular risk factors, the single diseases and factors are listed*.

* *some patients had more than one cardiovascular disease*.

** *some patients had more than one additional cardiovascular risk factor*.

At baseline, mean office systolic BP of the entire group was 161 ± 13 mmHg and the mean diastolic BP was 95 ± 8 mmHg. In comparison to patients with uncontrolled hypertension, those with untreated hypertension had a higher baseline systolic (163 ± 12 vs. 159 ± 13 mmHg; *p* < 0.001) and diastolic (97 ± 8 vs. 93 ± 9 mmHg; *p* < 0.001) BP. Based on the guidance by treating physicians, mean individual BP goals were set at 136 ± 6 mmHg for systolic BP and 84 ± 5 mmHg for diastolic BP.

### BP Treatment

Among the 289 patients with untreated hypertension at baseline, 187 (65%) were prescribed irbesartan 150 mg plus 12.5 mg HCTZ, 75 (26%) irbesartan 300 mg plus 12.5 mg HCTZ, and 27 (9%) irbesartan 300 mg plus 25 mg HCTZ as first line therapy.

Among the 491 patients with uncontrolled hypertension at baseline, 181 (37%) were prescribed irbesartan 150 mg plus 12.5 mg HCTZ, 176 (36%) irbesartan 300 mg plus 12.5 mg HCTZ, and 134 (27%) irbesartan 300 mg plus 25 mg HCTZ as replacement therapy (*p* < 0.001 for untreated vs. uncontrolled hypertension at baseline).

Prescription of increasing dose of irbesartan plus HCTZ was associated with increasing age (*p* = 0.001) and body weight (*p* < 0.001) but not with systolic and diastolic BP at baseline.

### BP Reduction, Goal Attainment, and Tolerability

**Figure 1:**
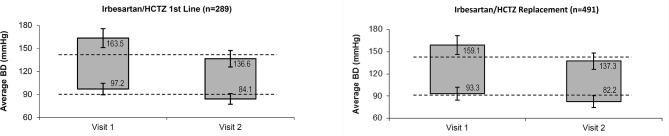
Average systolic and diastolic BP at baseline and follow-up visit. BP, Blood Pressure; HCTZ, Hydrochlorothiazide.

At the follow-up visit, mean systolic BP was 137 ± 11 mmHg and mean diastolic BP was 83 ± 8 mmHg. Mean reductions in systolic/diastolic BP after 8 weeks were 23.7 ± 13.7/11.7 ± 8.5 mmHg vs. baseline, with a reduction of 26.9 ± 14.1/13.0 ± 8.8 mmHg or 21.8 ± 13.1/11.0 ± 8.3 mmHg when the single-pill combination was given as first line or replacement treatment, respectively (*p* < 0.001 for difference between first line and replacement treatment in both systolic and diastolic BP). The development of the mean average BP is displayed in Figure 1.

In total, the guidelines-recommended goals for systolic BP were reached in 368 (47%; 95% CI 44–51%) patients, for diastolic BP in 492 (63%; 95% CI 60–66%), and for both systolic and diastolic BP in 312 (40%; 95% CI 37–43%). There was a higher goal attainment in systolic BP when the single-pill combination of irbesartan and HCTZ was chosen as first line vs. replacement treatment (52 vs. 44%; *p* = 0.043) (Table 2). The rate of goal attainment in diastolic BP was similar when the single-pill combination of irbesartan and HCTZ was chosen as first line vs. replacement treatment (66 vs. 62%; *p* = 0.24), and the same was true for goal attainment in both systolic and diastolic BP (43 vs. 38%; *p* = 0.20).

**Table 2:** BP goal attainment in first line and replacement single-pill combination therapy containing HCTZ.

	**Replacement therapy** ***N*** **=** **491**		**First line therapy** ***N*** **=** **289**		**Total** ***N*** **=** **780**		***P***
Systolic BP goal attainment, *n* (%)	218	(44.4)	150	51.9	368	47.2	0.043
Diastolic BP goal attainment, *n* (%)	302	(61.5)	190	65.7	492	63.1	0.24
Both systolic and diastolic BP goal attainment, *n* (%)	188	(38.3)	124	42.9	312	40.0	0.20

*BP, Blood Pressure; HCTZ, Hydrochlorothiazide*.

During the study period, 145 adverse events were recorded. Of those, 82 (57%) cases were classified as headache, 62 (43%) dizziness, 12 (8%) hypotension, 9 (6%) hypokalaemia, and 3 (2%) hyperkalaemia. Some patients had more than one adverse event. There was no difference in the occurrence of adverse events when the single-pill combination of irbesartan and HCTZ was chosen as first line or replacement treatment, besides a lower incidence of headache (8 vs. 12%; *p* = 0.043) (Table 3). Overall, compliance with study medication was rated as 100% in 368 (47%) patients, 80% in 286 (37%), 50–80% in 69 (9%), and 50% in 13 (2%), and was not reported in 44 (5%).

**Table 3:** Adverse events in first line and replacement single-pill combination therapy containing HCTZ.

	**Replacement therapy** ***N*** **=** **491**		**First line therapy** ***N*** **=** **289**		**Total** ***N*** **=** **780**		***P***
**Any adverse event**, ***n*** (%)^*^	**99**	**(20.2)**	**46**	**15.9**	**145**	**18.6**	**0.14**
Headache, *n* (%)	60	(12.2)	22	7.6	82	10.5	0.043
Dizziness, *n* (%)	37	(7.5)	25	8.7	62	7.9	0.58
Hypotension, *n* (%)	8	(1.6)	4	1.4	12	1.5	0.79
Hypokalaemia, *n* (%)	7	(1.4)	2	0.7	9	1.2	0.35
Hyperkalaemia, *n* (%)	2	(0.4)	1	0.3	3	0.4	0.89

*HCTZ, Hydrochlorothiazide. Bold identified total cardiovascular diseases and total cardiovascular risk factors, the single diseases and factors are listed*.

* *some patients had more than one adverse event*.

## Discussion

Irbesartan/HCTZ combination therapy used as both first line and replacement treatment were highly effective in lowering BP in patients with stage II and III hypertension. The response to treatment was rapid and interestingly, a larger reduction in BP was reached in previously untreated patients compared to patients who had responded inadequately to prior antihypertensive treatment, confirming the current recommendation of rather aggressive combination therapy as the preferred option for initial treatment of stage II or III hypertension (4, 5, 7, 16). However, even in patients previously treated the extent of BP lowering was substantial, suggesting perhaps some adherence issue to prior therapy. As a result of the efficient reduction in BP and good tolerability, many more patients may be able to reach the suggested BP targets when treated with the single-pill combination of irbesartan/HCTZ. The treatment of hypertension should be performed with the aim of attaining BP goals relatively rapidly, since significant delays or failure to achieve goals are associated with an increased risk of cardiovascular events in high risk patients (19). Since patients with moderate or severe hypertension are likely to require two or more agents to reach BP goals, the current recommendations of the Swiss, European, and American Societies of Hypertension are to initiate therapy with a single-pill combination in these patients (15, 18, 20).

The FLASH study involving 780 patients investigated the efficacy and safety of a single-pill combination BP-lowering therapy including HCTZ and irbesartan in subjects with moderate or severe hypertension, one third previously untreated and two thirds in whom prior therapy had not been sufficient to reach the target BP. The clinical profile of the study participants was balanced and in line with the patient characteristics from the CoLaus study, and it may thus be considered representative of the Swiss population of patients with hypertension (21, 22).

Not surprisingly, patients with uncontrolled hypertension despite prior antihypertensive therapy had a lower BP at baseline than previously untreated patients, were older and characterized by a higher proportion of clinically confirmed cardiovascular disease and additional cardiovascular risk factors (except of smoking). In such a population, adherence to medication is often an issue and the use of fixed-dose drug combination therapies is particularly helpful to reduce the pill burden (23).

Safety and tolerability, including hypotension, syncope, headache, and hypokalaemia are often used as an argument against aggressive antihypertensive therapy. In the present study, the single-pill combinations of irbesartan/HCTZ were well-tolerated (15, 16, 18, 20). Some treatment-related effects, such as hypotension and change in kalaemia with irbesartan/HCTZ combination therapy were observed, but none of these side effects was described as severe and there was no excess of withdrawals due to these side effects. Headache was frequent in our study. In the absence of a placebo group, it is difficult to judge how often headache was due to the combination of irbesartan and hydrochlorothiazide. In previous surveys, the incidence of headache was lower with angiotensin receptor antagonists than with placebo (24). A review published in 2001 reported a 30% incidence of side effects with different ARBs (25). As such, the incidence of side effect found in our study is less than reported in the literature, confirming that irbesartan and the combination of irbesartan and HCTZ are well-tolerated.

These findings are re-assuring and confirm the approach of the Swiss, European, and American Society of Hypertension in recommending aggressive two-drug therapy as initial treatment for patients with moderate or severe hypertension, and suggesting that this strategy can be pursued in a real-life setting without compromising patient safety (15, 16, 18, 20).

Our results are also consistent with other studies evaluating the effect of the fixed-dose combination of irbesartan/HCTZ (12). The benefits and good tolerability of irbesartan/HCTZ combination therapies have been demonstrated in a number of trials in patients with mild hypertension who failed to achieve BP control with monotherapy, as well as in patients with moderate or severe hypertension, renal failure, and in hypertensive type II diabetics with early or late-stage diabetic nephropathy (10, 26–28). Tolerability and the use of fixed-dose drug combinations are important factors impacting on patient's adherence and therefore on achievement of BP targets (23).

The present study has several limitations: First, the study was observational and the follow-up was limited to a short-term period. Second, the choice of antihypertensive therapy was not determined by an experimental plan but solely as the result of the treatment decision by the physician in charge. Third, we cannot exclude considerable inclusion bias because participating physicians had to enroll up to 6 patients only and may have selected those who were likely to respond best although many of them were uncontrolled. Due to lack of randomization and lack of a placebo arm in our study the results are to be considered hypothesis generating, not confirmatory, and therefore must be interpreted with caution. In contrast to randomized controlled trials, our study had no exclusion criteria and the results reflect the contemporary clinical practice. Finally, the study was performed in Switzerland only and the results may not be applicable to other regions.

In conclusion, the FLASH study suggests that irbesartan/HCTZ combination therapy is effective and well-tolerated in a population of patients with moderate or severe (stage II or III) hypertension either untreated or uncontrolled with previous therapy. The use of the single-pill combination of irbesartan/HCTZ as first line or replacement treatment achieved substantial reductions in both systolic and diastolic BP, with greater BP reductions when the combination therapy was chosen as first line vs. replacement treatment. The therapy was well-tolerated, and nearly half of the patients reached the guidelines-recommended BP target of <140/90 mmHg.

## Data Availability Statement

The raw data supporting the conclusions of this article will be made available by the authors, without undue reservation, to any qualified researcher.

## Ethics Statement

All subjects have given their written informed consent and the study protocol was approved by the research institute's committee on human research.

## Author Contributions

DS, MB, and GN were responsible for the study conception and design. DS and IS drafted the manuscript. SN, MB, SR, and GN critically revised the manuscript for intellectual content. All authors were involved in the data interpretation, contributed to the submitted work, and approved the final version of the manuscript.

### Conflict of Interest

The authors declare that this study received funding from Sanofi-Aventis (Suisse) SA, Vernier, Switzerland. The funder was involved in the study design and engaged in interpretation of data, writing of this article, and the decision to submit the manuscript for publication. The funder was not involved in the collection, management, and analysis of data. Data collection and data management were conducted by an Independent Clinical Research Organization. IS received speaker fee and research support by Sanofi-Aventis (Suisse) SA, Vernier, Switzerland. DS is an employee of Sanofi-Aventis (Suisse) SA, Vernier, Switzerland. MB received speaker fee by Sanofi International. The remaining authors declare that the research was conducted in the absence of any commercial or financial relationships that could be construed as a potential conflict of interest.
